# Within-subject effects of standardized prosthetic socket modifications on physical function and patient-reported outcomes

**DOI:** 10.1186/s13063-022-06205-z

**Published:** 2022-04-12

**Authors:** William Anderst, Goeran Fiedler, Kentaro Onishi, Gina McKernan, Tom Gale, Paige Paulus

**Affiliations:** 1 Department of Orthopaedic Surgery, University of Pittsburgh, Pittsburgh, USA; 2 Department of Rehabilitation Science and Technology, University of Pittsburgh, Pittsburgh, USA; 3 Human Engineering Research Laboratory, University of Pittsburgh, Pittsburgh, USA

**Keywords:** Limb prosthetics, Motion analysis, Dynamic radiography, Randomized controlled trial

## Abstract

**Background:**

Among the challenges of living with lower limb loss is the increased risk of long-term health problems that can be either attributed directly to the amputation surgery and/or prosthetic rehabilitation or indirectly to a disability-induced sedentary lifestyle. These problems are exacerbated by poorly fit prosthetic sockets. There is a knowledge gap regarding how the socket design affects in-socket mechanics and how in-socket mechanics affect patient-reported comfort and function. The objectives of this study are (1) to gain a better understanding of how in-socket mechanics of the residual limb in transfemoral amputees are related to patient-reported comfort and function, (2) to identify clinical tests that can streamline the socket design process, and (3) to evaluate the efficacy and cost of a novel, quantitatively informed socket optimization process.

**Methods:**

Users of transfemoral prostheses will be asked to walk on a treadmill wearing their current socket plus 8 different check sockets with designed changes in different structural measurements that are likely to induce changes in residual limb motion, skin strain, and pressure distribution within the socket. Dynamic biplane radiography and pressure sensors will be used to measure in-socket residual limb mechanics. Patient-reported outcomes will also be collected after wearing each socket. The effects of in-socket mechanics on both physical function and patient-reported outcomes (aim 1) will be assessed using a generalized linear model. Partial correlation analysis will be used to examine the association between research-grade measurements and readily available clinical measurements (aim 2). In order to compare the new quantitative design method to the standard of care, patient-reported outcomes and cost will be compared between the two methods, utilizing the Wilcoxon-Mann-Whitney non-parametric test (aim 3).

**Discussion:**

Knowledge on how prosthetic socket modifications affect residual bone and skin biomechanics itself can be applied to devise future socket designs, and the methodology can be used to investigate and improve such designs, past and present. Apart from saving time and costs, this may result in better prosthetic socket fit for a large patient population, thus increasing their mobility, participation, and overall health-related quality of life.

**Trial registration:**

ClinicalTrials.gov NCT05041998. Date of registration: Sept 13, 2021.

## Administrative information

Note: The numbers in curly brackets in this protocol refer to SPIRIT checklist item numbers. The order of the items has been modified to group similar items (see http://www.equator-network.org/reporting-guidelines/spirit-2013-statement-defining-standard-protocol-items-for-clinical-trials/).

**Table:** 

Title {1}	Within-subjects effects of standardized prosthetic socket modifications on physical function and patient-reported outcomes
Trial registration {2a and 2b}.	Registered on ClinicalTrials.gov: NCT05041998
Protocol version {3}	Version 1.0, March 26, 2021
Funding {4}	US Department of Defense, Award No: W81XWH2010914
Author details {5a}	William Anderst – Department of Orthopaedic Surgery, University of Pittsburgh, Goeran Fiedler – Department of Rehabilitation Science and Technology, University of Pittsburgh, Kentaro Onishi - Department of Orthopaedic Surgery, University of Pittsburgh, Gina McKernan – Human Engineering Research Laboratory, University of Pittsburgh, Tom Gale - Department of Orthopaedic Surgery, University of Pittsburgh, Paige Paulus - Department of Orthopaedic Surgery, University of Pittsburgh
Name and contact information for the trial sponsor {5b}	Tony L. Story Research and Development Analyst Congressionally Directed Medical Research Programs Office US Army Medical Research and Development Command 301-619-7033 (office) 240-344-4184 (mobile)
Role of sponsor {5c}	The sponsor has no role in the study design; collection, management, analysis, and interpretation of the data; writing of the report; and decision to submit the report for publication.

## Introduction

### Background and rationale {6a}

#### Importance of the problem

People with lower limb loss are at risk for a variety of chronic health challenges including cardiovascular diseases, osteoarthritis, low-back pain, obesity, and depression [1, 2]. The nature of those comorbidities differs substantially between patients who underwent amputation surgery as a consequence of vascular disease, such as diabetes, and those who experienced a traumatic amputation.

Among the civilian population, vascular disease is the predominant cause of limb loss [3]. Patients are typically older [4] and have a long history of disease manifestations involving the limbs, which include claudication, neuropathy, and tissue necrosis. The debilitating effects of these can contribute to a reduced activity level, sedentary lifestyle, obesity, and associated poor overall health. Because amputation surgery does not cure the underlying systemic diseases in this population, it is generally a last resort to address life-threatening limb complications. Even so, life expectancy after the surgery is limited [5, 6], and rehabilitation goals realistically are modest. Frequently, patients will not regain walking ability and remain constrained to wheeled mobility aids [7]. Prosthetic technology, if it is considered, generally reflects the limited rehabilitation goals in that prescribed functional components emphasize safety over dynamic efficiency and that prosthetic sockets are designed to be comfortable and easy to use at the expense of rigid suspension and controllability.

Limb loss after trauma is less prevalent in the overall population but is disproportionally common in the military population. While rehabilitation after combat-related amputations comes with its own set of challenges [8], including severe accompanying conditions such as complicated fractures, volumetric muscle loss, and traumatic brain injuries, the basic physical health of the patient is generally much better than in the average dysvascular amputation candidate [9]. This, along with the comparably young age of patients, calls for much more ambitious rehabilitation goals, including the full reconstitution of physical performance, participation in social life and gainful employment, and return to active duty [10]. Prescribed prosthetic components, such as knees and foot/ankle units are designed to support dynamic activity. The prosthetic socket may likewise be designed to be more functional and less comfortable, for instance, by providing a rigid ischial containment to prevent relative motion between the residual limb and prosthesis.

While people with traumatic limb loss are less at risk for sequela of a disability-induced sedentary lifestyle, they are nonetheless hampered by acute and chronic prosthesis-related conditions such as residual limb skin problems, low back pain, and osteoarthritis in the contralateral limb due to altered and excessive tissue loading during gait [11–13]. These problems are exacerbated by the limitations inherent in trying to reconcile the different objectives of prosthetic socket fit. There exists a belief among prosthetists that limiting skin strain and residual femur motion within the socket can minimize primary and secondary pathologies related to prosthesis use, and therefore, a prosthetic socket will always require unphysiological tight skin contacts and localized pressures. It has been proposed to avoid sockets entirely and attach prosthetic limbs directly to the residual bone using osseointegrated implants. Despite the great advancements that have been achieved with this approach in recent years [14], osseointegration may not be indicated for many individuals, either due to bone composition and co-morbidities [15] or to its incompatibility with high-impact activities such as running and sprinting [16] that military amputees may experience in the field. The more viable approach instead is to improve socket prostheses for this patient population.

#### Critical needs

People with lower limb loss experience chronic health challenges such as residual limb skin problems, low back pain, and osteoarthritis due to altered and excessive tissue loading during gait [11–13]. These problems are exacerbated by poorly fit prosthetic sockets. The goal for prosthetists in optimizing socket design is to improve function with a minimum of added discomfort. However, achieving an optimal balance between function and discomfort is currently a long, iterative process that is further frustrated by the absence of quantitative data [17, 18]. Instead, subjective patient feedback and coarse outcome assessments guide the repeated modifications. The lack of objective data also limits one’s ability to predict long-term outcomes (e.g., prosthesis-related tissue degradation).

**Fig. 1:**
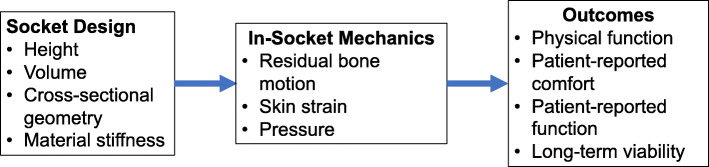
Key socket design parameters, in-socket mechanical factors, and patient outcomes

There is a knowledge gap regarding how the socket design affects in-socket mechanics and how in-socket mechanics affect patient-reported comfort and function (Fig. 1). Prosthetists believe that limiting residual femur and skin motion will improve socket function. However, there are no data to (1) indicate how socket design is related to these variables, (2) prescribe changes in a socket that will reduce these movements, and (3) confirm that reductions in residual femur and skin motion will improve patient-reported outcomes [19–22]. Consequently, there is a real need for an experimental approach to better understand the relationship between socket design and the dynamic response of the residual limb within the socket.

Although there are many types of prosthetic sockets marketed for transfemoral amputees, all are typically designed from a cast of the residual limb, do not account for soft tissue deformation and focal regions of high loading that develop during dynamic activities such as walking, and eventually lead to problems such as pressure ulcers, chronic skin problems, excessive joint loading, and an overall decreased quality of life for the amputee [23, 24].

Basic socket design principles have evolved steadily throughout the history of prosthetics and continue to be the subject of innovation. In trans-femoral prosthetics alone, a fairly large variety of basic design concepts are being discussed and marketed today, including varieties of ischial containment sockets (CAT-CAM, MAS) [25, 26], sub-ischial brimless designs (NUFlex, MWK) [27], “bionic” interface (HiFi, PBSS) [28], and adaptable frame designs (Infinite, Socket-less socket) [29], all claiming to entail substantial benefits and being widely indicated across the patient population. There is no scientific data and very little reliable empirical data to guide a choice of lower limb prosthesis from the available options. Because of this, any useful experimental approach for evaluating socket design on a patient-specific basis must be compatible with a patient group that uses a variety of different types of prosthetic sockets.

The socket optimization process involves refining the socket to optimize fit and function on a patient-specific basis. Socket optimization is an iterative process and dependent upon the experience and preferences of the prosthetist. There are some generally agreed upon (and taught) principles in optimizing socket fit, including, for example, local or general modifications of the socket volume, which are based on the observed difficulty of donning, skin discolorations, and patient-reported pressure points. The commonly applied process entails an iterative adjustment and reassessment of socket fit until a mutually (by patient and prosthetist) agreeable solution has been reached. However, there are many different approaches to such volume modifications. If a patient complains about a local pressure point, one prosthetist may decide to widen the socket in the respective area to relieve that pressure. Another prosthetist may narrow the socket in the area around the problematic spot in an effort to properly redistribute the contact force. Yet another may reduce the volume in the distal zones of the socket and reduce the length in order to reduce vertical displacement that, along with the conical shape of the limb, may originally have caused the intolerable contact pressure. The individual course of action thus depends on the suitability of the used assessment methods, the prosthetist’s interpretation of the issue, and his or her preference and experience.

#### Prior research

**Fig. 2:**
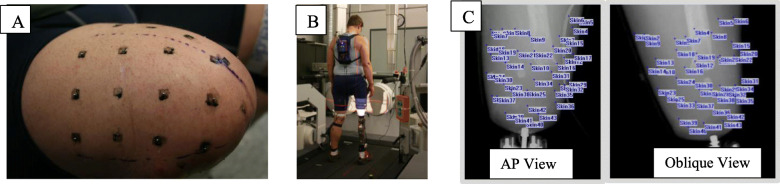
Method for measuring skin strain. **A** Metal beads were placed in a grid pattern on the skin of the residual femur. **B** Synchronized biplane radiographs were collected during treadmill walking. **C** Each bead was identified on every pair of synchronized radiographs to measure skin deformation during gait

We recently completed a study on 12 transfemoral amputees that demonstrates our abilities to complete the proposed study [30, 31]. In that study, we used dynamic biplane radiography to measure residual bone motion and skin deformation within the socket during gait for three steps in each of the12 transfemoral amputees. Dynamic biplanar radiography is an ideally suited technology for these purposes because it provides extremely accurate data (submillimeter accuracy) that can be collected at a high frequency (up to 150 images per second) to image motion within the socket during dynamic functional activities such as gait. Prior to testing, small (1–2 mm diameter) metal beads were secured to the skin of the residual limb prior to donning the socket (Fig. 2A). The participants then walked on a treadmill while biplane radiographs of the residual limb were collected at 100 images per second (Fig. 2B). The beads were identified in each pair of synchronized radiographs (Fig. 2C).

**Fig. 3:**
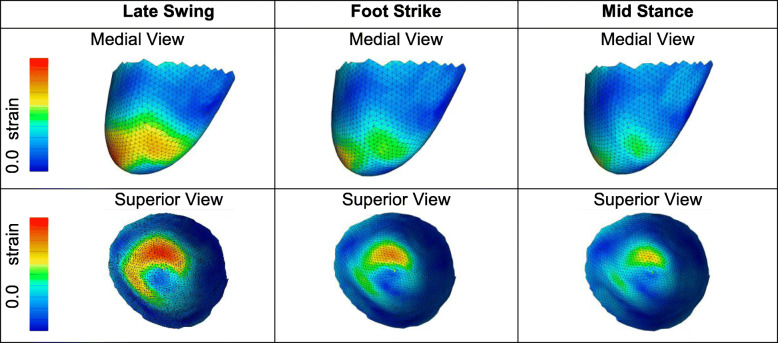
Skin strain within the socket during three instants of the gait cycle: late swing, foot strike, and midstance. The skin surface is color-coded according to skin deformation (strain). Note the region of high strain in the anterior-distal portion of the residual limb

We measured the relative motion between beads as the skin deformed within the socket for each participant. Skin deformation (strain) was then calculated from late swing through the push-off phase of gait (Fig. 3).

**Fig. 4:**
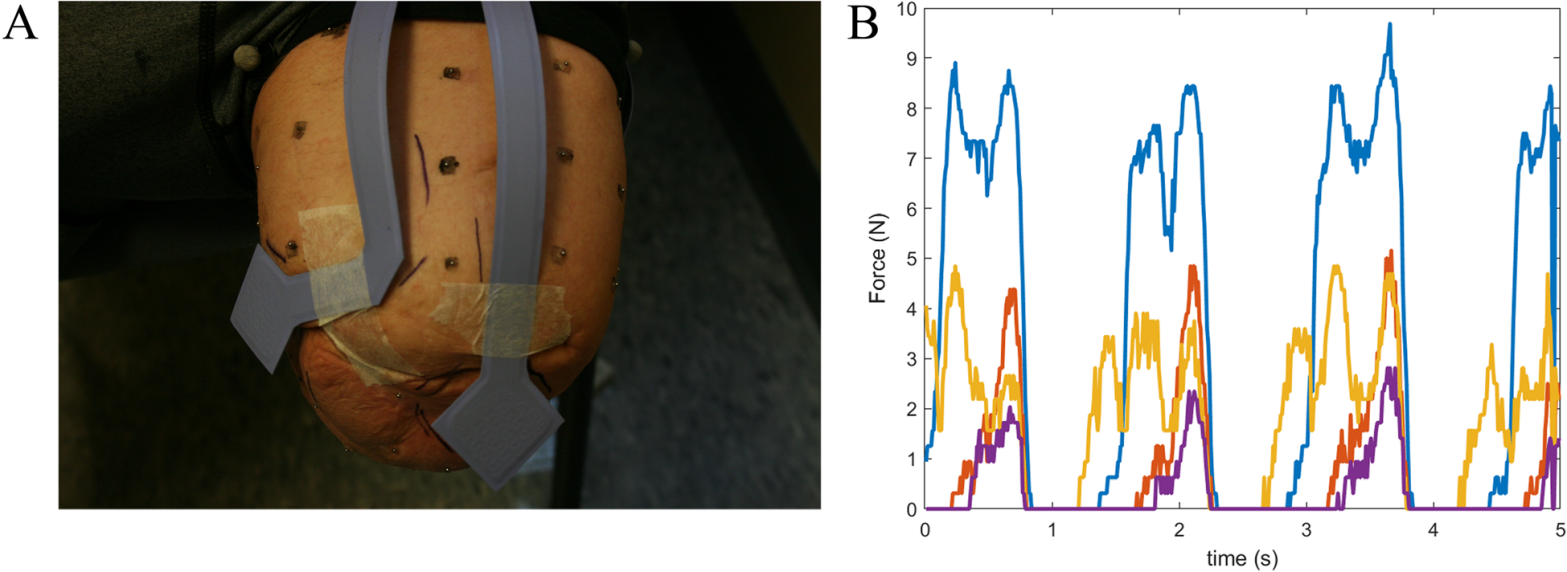
In-socket pressure measurement. **A** Pressure sensors were attached to the skin prior to donning the socket. **B** In-socket pressure was measured during gait. Each colored line represents the total pressure recorded by one of four sensor pads during 5 s of gait

The feasibility of measuring in-socket pressure during gait was also successfully demonstrated on one subject (Fig. 4).

**Fig. 5:**
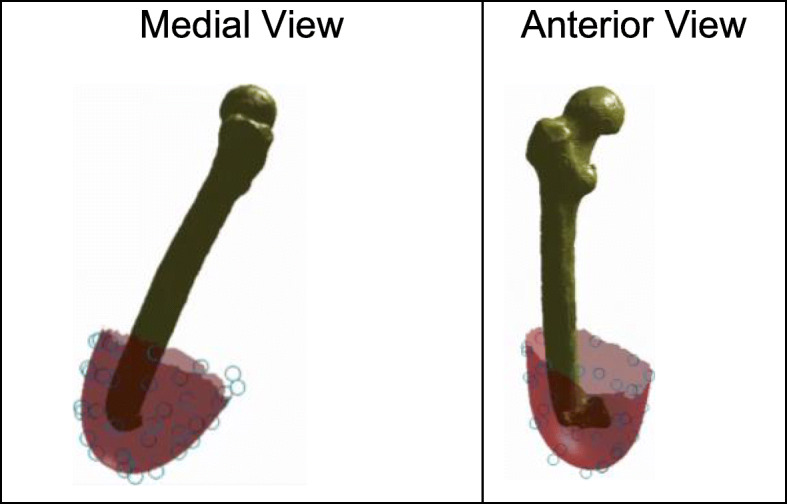
Residual femur position within the socket of a transfemoral amputee at late swing during the gait cycle

After lab testing, a CT scan of the residual femur was collected. The bone tissue was segmented from the CT scan and used to create a patient-specific 3D model of the residual femur. We then used our validated model-based tracking system to measure the motion of the residual femur within the socket by matching the femur bone model to the synchronized biplane radiographs (Fig. 5).

That study demonstrated feasibility for this proposed study, allowed us to refine our data collection and analysis methods, and provided novel data to characterize the dynamic in-socket mechanics of transfemoral amputees. Over the course of that study, we developed a method to secure markers onto the skin in a grid pattern with a sufficient number of markers to obtain smooth, continuous skin deformation measurements. Importantly, none of the patients reported discomfort during gait due to the small markers secured to their skin. We were also able to identify an imaging configuration for the biplane radiography system that allowed us to image the residual limb from late swing through push-off with limited or no occlusion from the contralateral leg in the biplane radiographs. One encouraging finding was the repeatability of the in-socket mechanics. Although each participant demonstrated unique skin and residual femur movement patterns, within participants these in-socket mechanics were repeatable from trial to trial. One unexpected finding was the magnitude and rate of skin strain during the late swing phase. We had anticipated the greatest skin strain would occur after foot strike; however, our findings suggest that the skin is stretched most rapidly during the late swing phase as the leg is extended just prior to foot strike and the prosthesis pulls on the residual limb. This period of high strain rate is then followed by less rapid skin deformation during the loading phase. Those findings highlight the value of measuring dynamic in-socket mechanics and have helped guide our experimental design for this proposed study.

### Objectives {7}

The objectives of this proposal are (1) to gain a better understanding of how in-socket mechanics of the residual limb in transfemoral amputees are related to patient-reported comfort and function, (2) to identify clinical tests that can streamline the socket design process, and (3) to evaluate the efficacy and cost of a novel, quantitatively informed socket optimization process.

Our scientific premise is that understanding the biomechanical interactions between the prosthetic socket and the residual limb is fundamental to improving socket design. In order to optimize socket fit and distribute dynamic loads, it is critical to understand how the residual limb tissues respond to the external loads during gait. We are proposing that the current socket optimization process, which focuses exclusively on the relationship between socket features and outcomes, can be improved and expedited by using a quantitatively informed iterative optimization process that can improve comfort and function and reduce long-term health effects along with the associated healthcare costs.

### Specific aims

This project is structured with three specific aims. Aim 1 will build a foundation by using research-grade tools and technology to identify the relationship between socket design, in-socket mechanics, and patient-reported comfort and function. Aim 2 will identify readily available clinical measurements that are associated with in-socket mechanics during dynamic activities. Findings from aims 1 and 2 will be used to develop a quantitatively informed design optimization process that will be evaluated and compared to the current standard of care in a pilot clinical trial as part of aim 3. The specific aims and associated hypotheses are as follows.

#### Specific aim 1: Determine the relationship between in-socket mechanics and patient-reported comfort and function

The purpose of this aim is to reveal how in-socket mechanics, that is, (1) residual femur motion relative to the socket, (2) skin strain, and (3) pressure distribution, are related to socket design and patient-reported comfort and function. We hypothesize that (H1) the amount of residual bone motion, (H2) the peak rate of skin strain, and (H3) the peak pressure within the socket are associated with patient-reported comfort and function.

#### Specific aim 2: Identify readily available clinical measurements that are associated with residual femur motion, skin strain, and peak pressure within the socket during dynamic activities

Being able to quantify the parameters of interest in a research setting does not easily translate to clinical application. The purpose of this aim is to correlate our laboratory findings with measurements obtained by conventional clinical assessments. We hypothesize that the following clinical measurements correspond to dynamic in-socket mechanics: (H1) static imaging under different loads will correspond to dynamic residual femur motion, (H2) gait analysis will correspond to dynamic pressure distribution, and (H3) residual limb tissue compliance will correspond to peak skin strain.

#### Specific aim 3: Develop and evaluate an improved socket design and optimization process through the use of a predictive model based upon clinical measurements

In order to generate preliminary evidence on the clinical utility of the findings from aims 1 and 2, we will perform a pilot trial, using new subjects, to compare our data-informed optimization process resulting from aim 2 to the conventional iterative process for selecting a socket design and then optimizing the socket fit and function. We hypothesize that the data-informed method is advantageous in terms of (H1) patient-reported outcomes and (H2) cost, defined by the number of visits and socket iterations, and the amount of labor required to optimize the socket fit and function.

### Trial design {8}

**Fig. 6:**
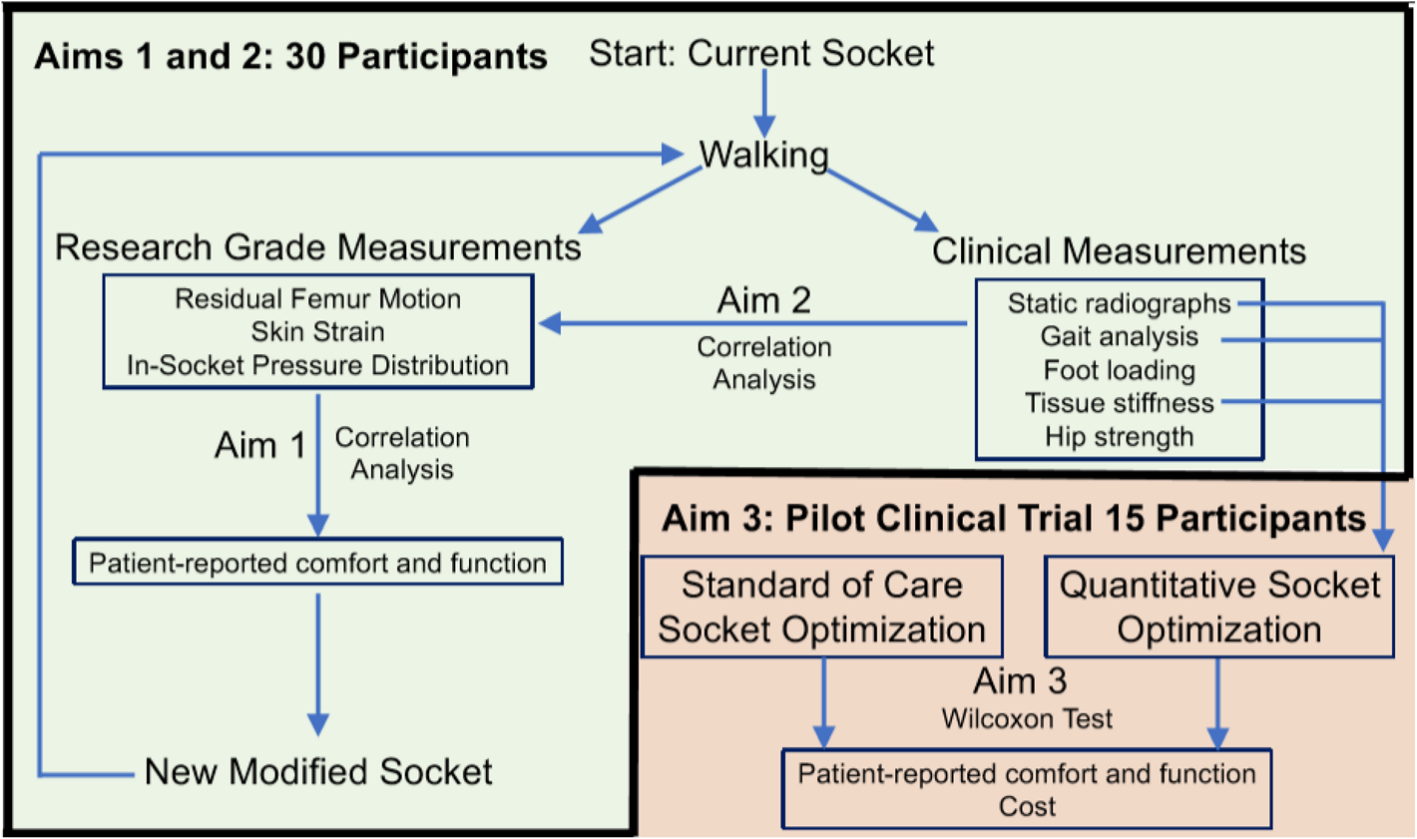
Flowchart showing the relationship between aims 1 and 2 (green background) and the pilot clinical trial, aim 3 (orange background). As an example, the flowchart shows three clinical measurements identified in aim 2 that correlate to in-socket mechanics obtained from research-grade measurements. In this example, those three clinical measures will then be used as predictors to guide the quantitative socket design

The trial design is an exploratory single-group crossover trial (Fig. 6).

## Methods: participants, interventions, and outcomes

### Study setting {9}

The study will be conducted at the Biodynamics Laboratory, an academic research laboratory within the University of Pittsburgh. Participants will be recruited from the local limb loss population.

### Eligibility criteria {10}

We aim to work with a representative, yet homogeneous sample of transfemoral amputees. All prosthesis users will have been ambulating with a prosthetic for at least 1 year. All participants will be under 125 kg of body weight. The amputation risk in males is 1.7 (dysvascular) to 4.9 (traumatic) times greater than in females [32], and our sample is assumed to be representative of that distribution. A weight limit of 125 kg is typical for most standard prosthesis componentry. Participants will be between 18 and 80 years of age and must be able to walk unassisted on a treadmill. Pregnant women will be excluded from the data collection to avoid the risk of exposure to ionizing radiation. An additional inclusion criterion for the participants in the pilot clinical trial is that they will be in need of a new socket.

The cause of limb loss will not be considered as an inclusion criterion, as the principal mechanisms under investigation apply irrespectively of the amputation cause. It may be possible to investigate any differences by etiology in post hoc analysis. Likewise, the residual limb length will not be considered as an inclusion criterion but will be documented and analyzed as appropriate post hoc.

### Who will take informed consent? {26a}

Participants’ informed written consent will be obtained by the study PI, Dr. Anderst, or the study coordinator.

### Additional consent provisions for collection and use of participant data and biological specimens {26b}

Not applicable as this trial does not involve collecting biological specimens.

### Interventions

#### Explanation for the choice of comparators {6b}

Every participant in aims 1 and 2 will be fitted with several different check-sockets for this study, each deliberately modifying one of the independent variables: brim height, volume, cross-sectional geometry, and material stiffness. These variables are known to have an effect on the comfort and function of the socket, and they are among those routinely altered in the static and dynamic fitting process [33]. Those modifications are generally iterative and performed in conjunction with others. For instance, changes in the geometry such as the oblique medio-lateral dimension at the perineum by padding the area of the ischial containment level will also affect the volume of the socket as well as the material stiffness in the modified region. The degree of modifications and manner of combinations is very much customized and informed by individual characteristics such as soft tissue content and pain tolerance. For the study, in order to standardize interventions as much as possible and minimize the risk of drastically compromised socket fit, we will make well-defined modest changes using the participants’ original (current) sockets as the baseline. One single prosthetist will facilitate all the modifications to assure consistency across the sample.

#### Intervention description {11a}

For all aims, we will start with a shape capture of the existing socket. From that scan, a positive model will be generated that will enable the fabrication of check sockets. Six to eight such check sockets will be produced for each participant as detailed below, using industrial service fabrication, where applicable, to make the variants. This will result in consistent socket quality across sockets and across participants.

##### Modified stiffness

Two check sockets based on the original positive model will be fabricated using different materials that represent different stiffness grades [34]: polyethylene terephthalate glycol (PETG), sold as Vivak®, and ethylene vinyl acetate (EVA), sold as ThermoLyn Soft®. Both material types are routinely used in clinical prosthetics, where the stiffer PETG material is preferred for check socket fittings and the softer EVA material for definitive sockets (where long-term dimensional stability can be supported by an additional laminated container socket).

##### Modified brim height

The PETG copy of the participants’ existing socket can be modified by grinding down the material to a desired lower brim height. Assuming that the socket fits well, there is generally no clinical rationale for raising the brim height because it would worsen both fit/function and comfort. Instead, the brim height will be reduced in two stages, by 5 and 10% of the overall socket length. Lowering the brim height (up to a certain point) will, by trend, increase wear comfort and hip range of motion but decrease suspension and weight-bearing ability. Depending on the randomization schedule for an individual participant, not all two versions of the socket with the lower brims will have to be fabricated, but the initial socket from above will be modifiable between tests to represent one or both of the other intervention levels.

##### Modified volume

Next, two derivative sockets will be fabricated (from PETG material) that are 6% oversized and undersized, by globally altering the model volume in accordance with established methods. Volume reductions of up to 6% from the original cast model are commonly undertaken in clinical prosthetics and are informed by the soft tissue content of the residual limb [17].

##### Modified cross-sectional geometry

**Fig. 7:**
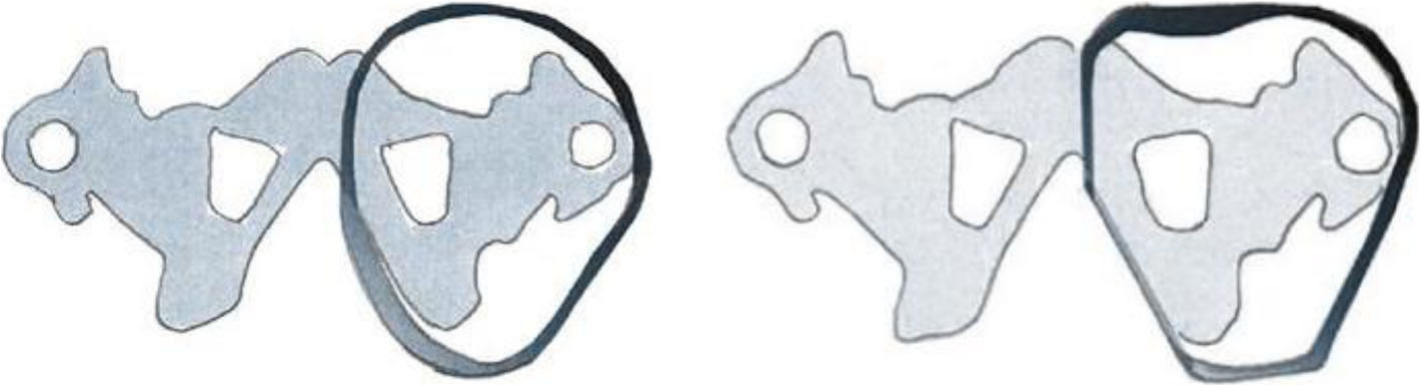
Illustration of geometry modifications. The CAT-CAM socket on the left follows widely the limb anatomy; the MAS socket on the right is more aggressive in defining the limb shape (adapted from Psonak, Richard. “Transfemoral prostheses” in orthotics and prosthetics in rehabilitation, Oxford, Elsevier, 2^nd^ Edition, 2007: 652-684)

Much like the socket volume reduction will be influenced by the tissue composition of the residual limb, the degree to which the socket geometry re-shapes the uncompressed limb will vary between patients. This can be illustrated when considering the differences between the classic ischial containment socket, termed Contoured Adducted Trochanteric-Controlled Alignment Method (CAT-CAM) by Sabolich [35], and the more recent Marlo Anatomical Socket (MAS), proposed by Ortiz [36] (Fig. 7). For the study, the original socket model of each participant will be the basis for two modifications of the cross-sectional geometry toward the opposite ends of the spectrum. Inevitably, the degree to which those modifications are possible without interfering with the usability of the socket is subject to individual differences and will be determined by the study prosthetist accordingly.

#### Criteria for discontinuing or modifying allocated interventions {11b}

Socket comfort will be routinely monitored as part of the outcome assessment during the intervention periods. Any adverse effects will be recorded, reported, and evaluated in correspondence with applicable rules. In the event that it is determined that an observed adverse event is attributable to the intervention (and not a unique response/unique circumstances in the individual case), this intervention will be discontinued or modified to address the issue for this and subsequent data collection sessions.

#### Strategies to improve adherence to interventions {11c}

For aims 1 and 2, interventions (modified check sockets) will be administered only during data collection sessions in the lab. Study personnel will be present throughout those periods to respond to or prevent any issues, including a lack of adherence (i.e., participants doffing the socket during the test).

For aim 3, participants will be fitted with a new socket that will replace their original one on their regular prosthesis. The original socket will be retained at the prosthetist’s office to prevent unauthorized switching back by the participant or others. Participants will be asked to not use other prostheses they may own during the study period, and adherence to this will be evaluated by exit interviews.

#### Relevant concomitant care permitted or prohibited during the trial {11d}

There are no interactions between the intervention and any concomitant care or interventions. Participants will be encouraged to make no adjustments to their normal routine while in the data collection protocol.

#### Provisions for post-trial care {30}

Participants will be able to follow up with their prosthetist, who is part of the study team, as part of their continued routine prosthetic care. Any long-term harms from participation can be assessed during these appointments. Due to the nature of the intervention (prosthetic socket modifications comparable to routine clinical care interventions), no long-term adverse effects are anticipated.

### Outcomes {12}

Outcomes for each participant will be averaged across three trials for each of the interventions, to analyze the change from baseline with different socket designs.

Many of the assessment methods are identical or equivalent to clinical assessment instruments that are associated with high validity and low risk for the target population. The effective radiation dose due to dynamic biplane radiography is estimated to be 2.0 mSv or less for the 27 trials collected for each participant. For comparison, the average effective dose associated with a knee CT scan from our previous studies was 1.4 mSv, and in the USA, we receive about 3.0 mSv of exposure from natural background radiation every year [37].

The following is the primary outcome variable:

- *Socket comfort*

The following are the secondary outcome variables:

- *Hip range of motion (ROM)*
- *Three-dimensional (3D) skin motion within the socket*
- *Residual femur position within each socket*
- *In-socket pressure*
- *Plantar pressure*
- *Ground reaction forces during gait*
- *Overall body motion (e.g., trunk lean, hip flexion/extension) during gait*

#### Participant timeline {13}

**Fig. 8:**
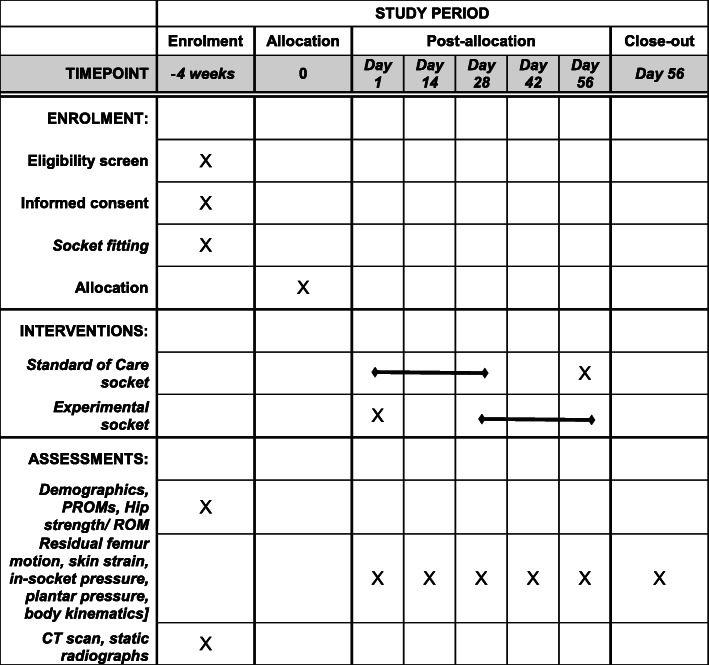
Schedule of enrollment, interventions, and assessments

Participants in the first stage (aims 1 and 2) of the study, upon eligibility screening and completing the consent process, will be fitted for a check socket. After 4 weeks, during which time 8 derivatives of the socket will be produced, participants will complete testing with each of those socket variants over one or two lab visits. The participant timeline for the second stage (aim 3) includes clinical testing of one socket variant and spans a larger period (Fig. 8).

#### Sample size {14}

To facilitate aims 1 and 2, GLM will be utilized, which is a flexible generalization of ordinary linear regression that allows for response variables that have error distribution models other than a normal distribution and is often robust to smaller sample sizes. With a sample size of *n* = 30 at 80% power and *α* = .05, we should be able to detect a moderate-to-large overall model effect (*f*^2^ = 0.45), while including all three in-socket mechanics. We will examine the effect(s) of each in-socket mechanical parameter on outcomes by utilizing the type III sum of squares tables. If our sample size drops below 30, we will develop separate models for each of the parameters of interest and outcome.

For aim 3, in order to compare the two methods of socket design (the quantitative design method compared to standard of care), we will compare patient-reported outcomes and cost between the two methods, utilizing the Wilcoxon-Mann-Whitney non-parametric test. With a sample size of *n* = 15 at 80% power and *α* = .05, we will be adequately powered to detect a moderate-to-large effect (*d* = 0.476) with respect to the differences in patient-reported outcomes and cost by method.

#### Recruitment {15}

Participants will be recruited from the local limb loss population. The investigators have access to a group of transfemoral amputees who have volunteered to serve as patients for students within the Orthotics and Prosthetics Program at the University of Pittsburgh, as well as participants of previous studies who were identified through support groups and word of mouth. Participants will also be recruited from the clinics of a local physiatrist and the prosthetics and orthotics practice of a collaborating local prosthetist. Further recruitment avenues include the Pitt+Me Research Registry comprising over 117,000 individuals who have volunteered to be contacted for research studies at the University of Pittsburgh, and social media including Facebook.

### Assignment of interventions: allocation

#### Sequence generation {16a}

The order of the modified sockets worn by each participant will be randomized using computer-generated random numbers.

#### Concealment mechanism {16b}

Data from each trial will be labeled with non-descriptive identifiers. The participant will not be told the modification made to each socket but will be allowed to see and touch the socket, as this is inevitable when using the prosthesis.

#### Implementation {16c}

The allocation sequence will be generated by the study statistician who is not involved in the data collection. All participants in either study phase will be enrolled by the study PI and will eventually be exposed to all interventions of the study phase in a randomized sequential order.

### Assignment of interventions: blinding

#### Who will be blinded {17a}

The research technician and engineer will be blinded to the socket modification when processing radiographic data from each trial, which will be labeled with non-descriptive identifiers. Effective blinding of trial participants, care providers, and data collection personnel will not be possible as the appearance of the prosthesis cannot be altered without affecting relevant characteristics (such as fit or weight).

#### Procedure for unblinding if needed {17b}

Upon conclusion of the analysis, participants and study personnel will be provided with the unblinding key (i.e., a list with the individual intervention sequence).

### Data collection and management

#### Plans for assessment and collection of outcomes {18a}

##### PROMs

Participants will be asked to rate their overall perception of the (1) comfort and (2) function of each socket relative to their current socket on a scale ranging from − (a very great deal worse) to 0 (about the same) to 7 (a very great deal better) using a global rating of change scale [38] (GRC). The GRC scale was selected over other questionnaires because, intuitively, it makes sense that a patient’s perception of comfort and function gives a more accurate assessment if a true change has occurred rather than a prognostic rating [39]. The Prosthetic Limb Users Survey of Mobility (PLUS-M) 12-item short form will be collected after wearing of each socket to provide a secondary measure of perceived mobility. In addition, participants will be completing the OPUS device satisfaction survey to establish baseline satisfaction with their current device and the Amputee Mobility Predicter with prosthesis (AmpPro), attached, to assess their mobility level. All of these data will be collected via tablet using the REDCap system, a secure, web-based application designed to optimize data collection. The REDCap system is currently in place and used to collect data for ongoing research studies in the Biodynamics Lab.

##### CT scans

We will acquire a CT scan of the residual femur from each participant. The scan will include a series of 0.625-mm slices from the proximal to the distal end of the residual femur.

##### Hip strength and ROM

**Fig. 9:**
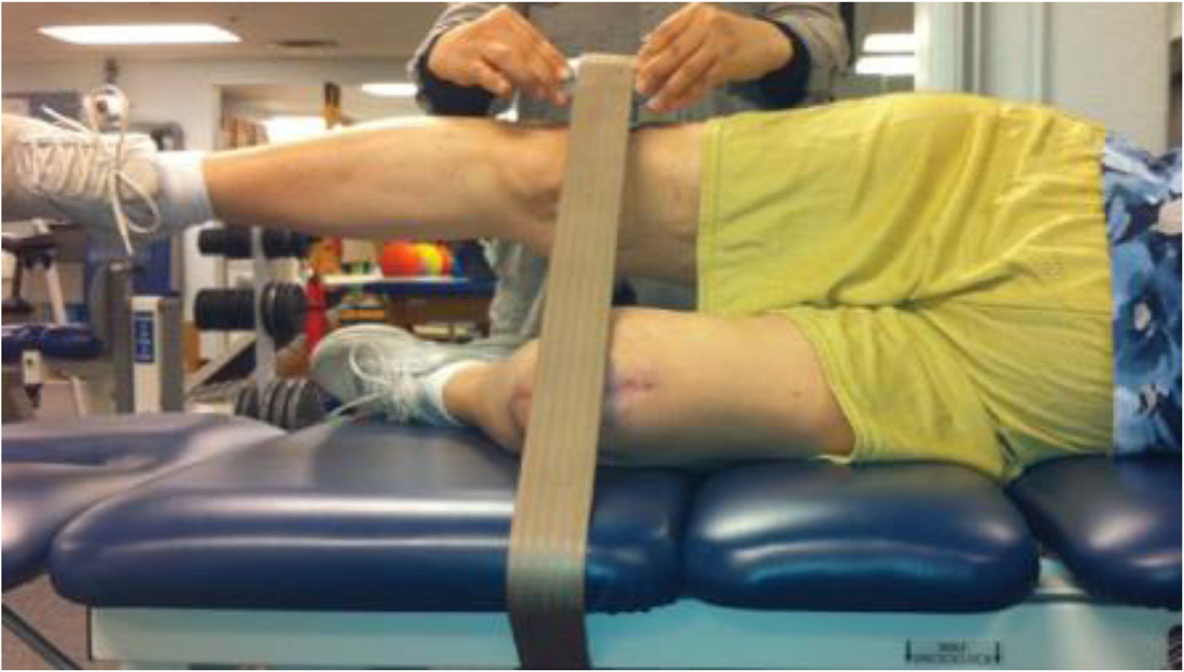
The hip abductor test

Hip abductor and flexor strength will be measured with a hand-held dynamometer (HHD) (Lafayette Instrument, Lafayette, IN) using previously established protocols [40] modified for amputees. Hip abductor strength will be measured with the participant side-lying on an examination table with the test hip facing up (Fig. 9). The non-test leg is bent at the hip and knee for stability. The test hip is in neutral flexion/extension and rotation. The moment arm is recorded as the distance in centimeters between the greater trochanter and the HHD. A non-elastic strap is used to secure the dynamometer against the leg and provide resistance to hip abduction. The strap is wrapped around the leg and underneath the table. The participant is asked to push up against the HHD as hard as possible for 4 s (substantial reliability; ICC .95) [41]. This procedure will be repeated to test hip flexor strength with the participant supine on the exam table and arms across the chest. Hip range of motion will be measured with a standard long-arm goniometer during active (moved by the participant) and passive (assisted by the tester) movements with the participant standing upright using crutches to balance.

##### Dynamic biplane radiography

**Fig. 10:**
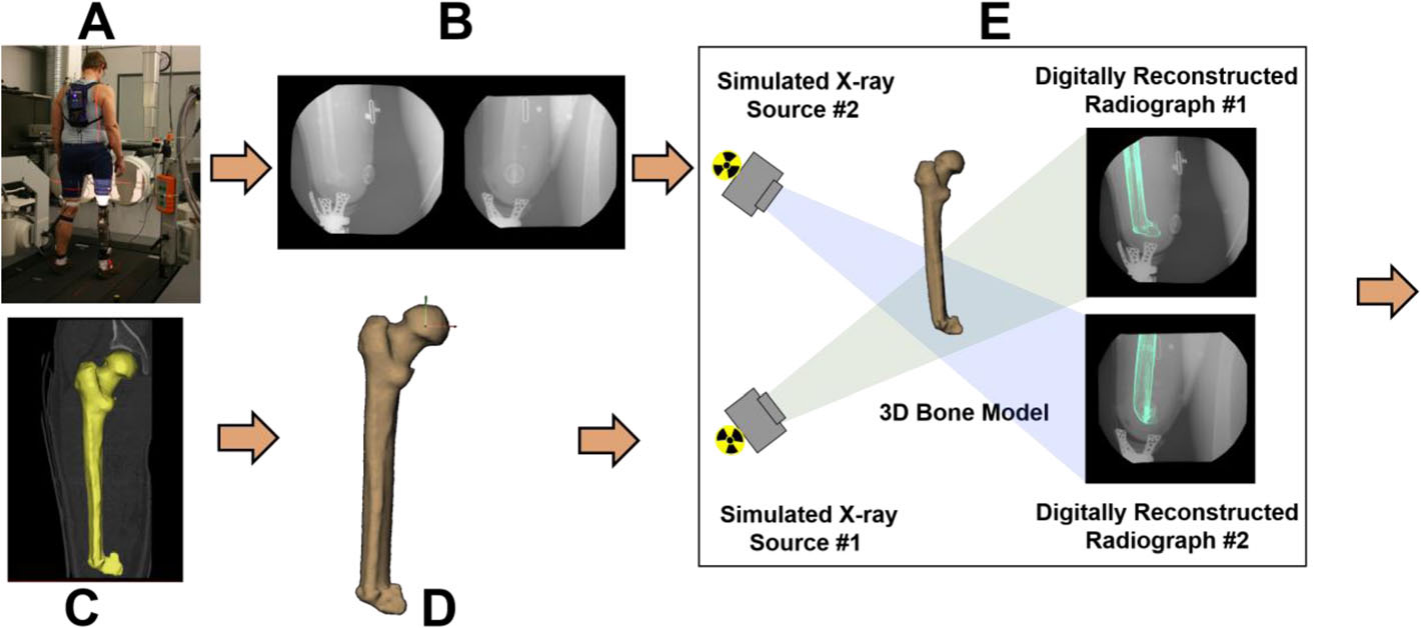
Biplane radiography data collection and processing. **A** Participants will walk on a treadmill while **B** synchronized biplane radiographs will be collected at 100 images per second (70 kV, 125 mA, 1 ms exposure per image). **C** CT scans of the residual femur will be collected and **D** used to create 3D bone models. **E** 3D bone pose will be determined using an automated volumetric model-based tracking process that matched digitally reconstructed radiographs to the original radiographs. **F** Six degree-of-freedom kinematics, including bone motion relative to the socket, will be calculated from late swing through push-off

Participants will then walk at their self-selected pace which will be held constant by the treadmill while wearing each of the 9 socket designs. We selected walking because it is the most common lower body functional activity, and comfort and function during walking have a major impact on amputee quality of life. After donning the socket, the participant will be allowed time to acclimate to each socket by walking on the treadmill for up to 5 min. After acclimation, we will collect a total of three trials for each of the 9 sockets. We will collect synchronized biplane radiographs for 1 s at 100 images per second to image the residual femur and skin motion within the socket during the late swing through push-off phase of gait. Images will be collected with 1-ms exposures in all cases to minimize motion blur and radiation exposure. Three-dimensional (3D) skin motion within the socket will be determined by tracking the motion of 40 to 50 small metal beads (1 to 2 mm diameter) that will be secured to the skin of the residual limb in a grid pattern before donning the socket (Fig. 2A). Beads will be secured using Quick Grip glue, a type of contact cement that does not permanently bond to the skin like Super Glue but is strong enough to hold the bead to the skin without moving. Residual femur motion will be determined with sub-millimeter accuracy using a validated volumetric model-based tracking process that matches subject-specific bone models obtained from CT to the biplane radiographs [42–44] (Fig. 10).

##### Static radiographs

A series of three static radiographic images of the residual femur within each socket will be collected under increasing load applied to the prosthetic side (25%, 50%, 100% bodyweight), with the load measured by the force plate imbedded within the treadmill.

##### In-socket pressure

**Fig. 11:**
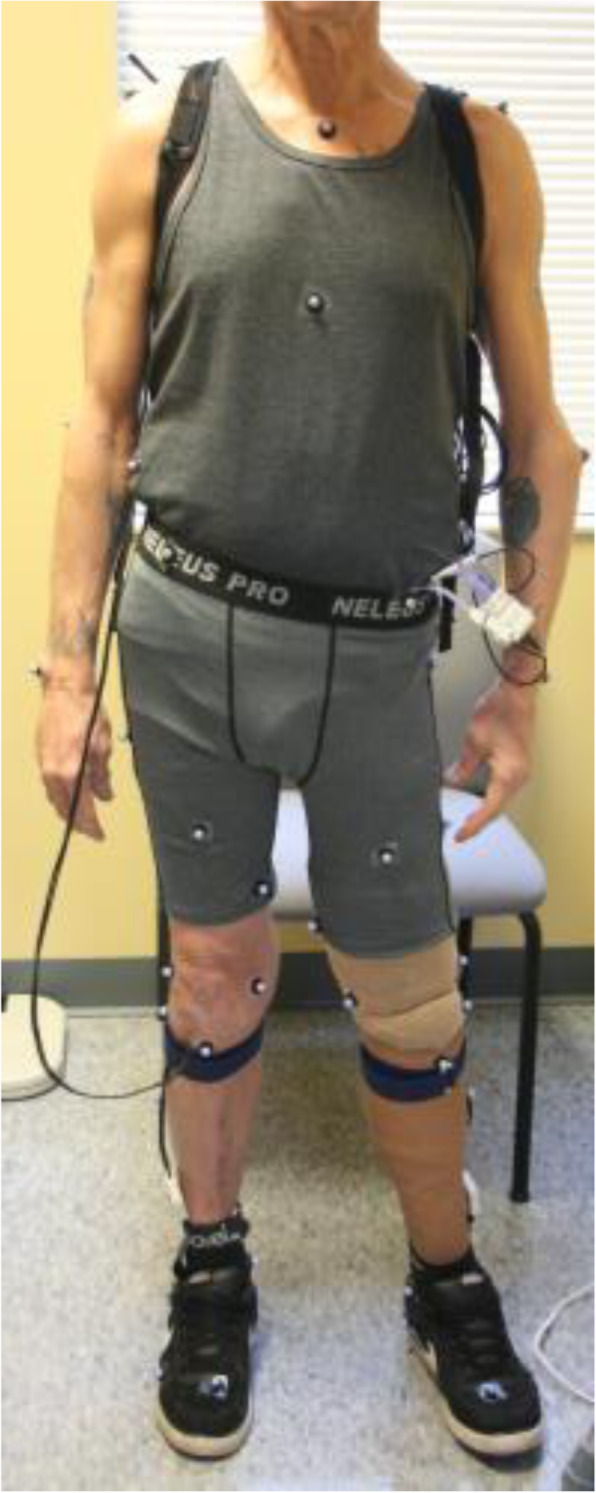
Reflective markers on a transfemoral amputee. Conventional motion capture will be used to measure overall body motion during gait

Pressure within the socket will be recorded at 65 Hz using 4 discrete pressure sensor pads, each less than 1 mm in thickness, each comprising 25 pressure sensors covering a 2 cm × 2 cm area (Pliance socket sensors, Novel Electronics, Inc.). The four pressure sensors will be placed in the anterior, posterior, medial, and lateral regions of the distal socket (Fig. 11).

##### Plantar pressure

Foot plantar pressure will be measured at 100 Hz using insoles comprising 99 pressure sensors (Pedar system, Novel Electronics, Inc.) for the prosthetic and contralateral foot during each step of gait. At least 6 consecutive steps will be collected for each walking trial for each socket design.

##### Ground reaction forces

Ground reaction forces will be collected at 1000 Hz using a dual-belt instrumented treadmill (Bertec Corp). This system contains two side-by-side 30 × 180 cm belts. The belts are driven by independent (but synchronized) motor systems, and each belt/motor system is configured on a rigid platform supported by multi-axis load cells. This configuration enables the assessment of three-dimensional foot-ground reaction forces, applied torque, and center of pressure location independently for each foot. Ground reaction force data will be collected primarily to determine foot strike (greater than 50 N total force) and toe off (less than 50 N total force) during the gait cycle.

##### Motion capture

Conventional motion capture will record overall body motion (e.g., trunk lean, hip flexion/extension) during gait at 200 Hz using a set of 53 reflective markers placed on the participant (Fig. 10) (12 cameras, Vicon MX). Webcams placed behind and to the side of the participant will record standard video (30 Hz) of each walking trial.

##### Ultrasound elastography

**Fig. 12:**
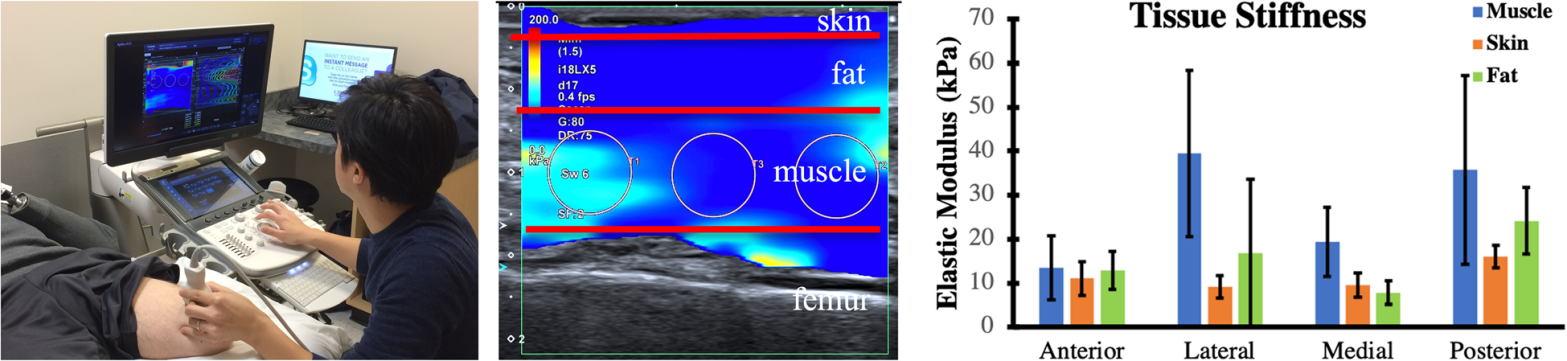
Ultrasound elastography performed on the residual limb of a transfemoral amputee (left). Tissue stiffness is color mapped onto the original ultrasound image and measured within 3 regions of interest (middle). Average values within each region are determined for each tissue (right)

Tissue stiffness in the anterior, posterior, medial, and lateral regions of the residual limb will be measured using ultrasound elastography [45]. Stiffness will be measured 7 cm proximal from the tip of the residual limb and separated into skin, fat, and muscle regions. Each measurement will comprise 4 regions of interest within each tissue and will be repeated three times. The average values of all measures will be used for analysis (Fig. 12).

##### Data processing and analysis

Residual femur bone tissue will be segmented from the CT images. All segmentation will be performed using the Mimics software (Materialise, Leuven, Belgium) using a combination of automated (thresholding and region growing) and manual segmentation strategies. We will measure the length of the residual femur and the bevel angle of the distal femur relative to the femur long axis.

##### Residual femur motion

Our validated volumetric model-based tracking system will match subject-specific 3D bone models to the biplane radiographs [42, 46] (Fig. 9). This tracking system has been validated in vivo to have an accuracy of 0.7 mm or better in translation and 0.9° or better in rotation [42] for 6 degree of freedom kinematics. Details describing the volumetric model-based tracking process, including hardware and software specifications, calibration and distortion correction procedures, and computational algorithms, have been described in detail [42, 47, 48]. The tracking process will yield the 3D motion of the residual femur during each walking trial. The dynamic 3D femur motion data will be smoothed using a fourth-order, low-pass Butterworth filter, with the filter frequency determined using residual analysis [49]. We will measure six degree of freedom motion of the femur relative to the socket (anterior-posterior, medial-lateral, and proximal-distal translation; anterior-posterior tilt, varus/valgus rotation, and internal/external rotation) and express our results as a percentage of the gait cycle. The analysis for aims 1 and 2 will focus on the medial-lateral and superior-inferior translation of the distal femur relative to the socket from late swing through midstance.

##### Skin strain

The motion of the beads attached to the skin will be tracked using a custom software to identify the center of each bead in each pair of synchronized radiographs. This software finds bead centroids with an accuracy of 0.08 to 0.12 mm during dynamic movements [42, 43]. Skin strain will be calculated using freely available software (FEBio), with the zero strain value determined while wearing the socket but not bearing weight. Variability in the zero strain measurement will be determined by doffing and donning the socket three times and imaging the residual limb after each donning. The residual femur will be divided into 4 primary regions (anterior, medial, posterior, and lateral), with proximal and distal sub-regions within each region, resulting in 8 total regions. The average and peak skin strain within each region will be determined and expressed as a percentage of the gait cycle. The average values over all steps will be included as variables in the analysis for aims 1 and 2.

##### In-socket pressure

We will measure peak pressure and area under the pressure versus the time curve recorded by each in-socket pressure sensor. Pressure will be measured for up to 6 steps per walking trial (i.e., 18 steps per socket) and the pressure results expressed as a percentage of the gait cycle (Fig. 4B). The average values over all steps will be included as variables in the aim 1 and aim 2 analysis.

##### Plantar pressure

**Fig. 13:**
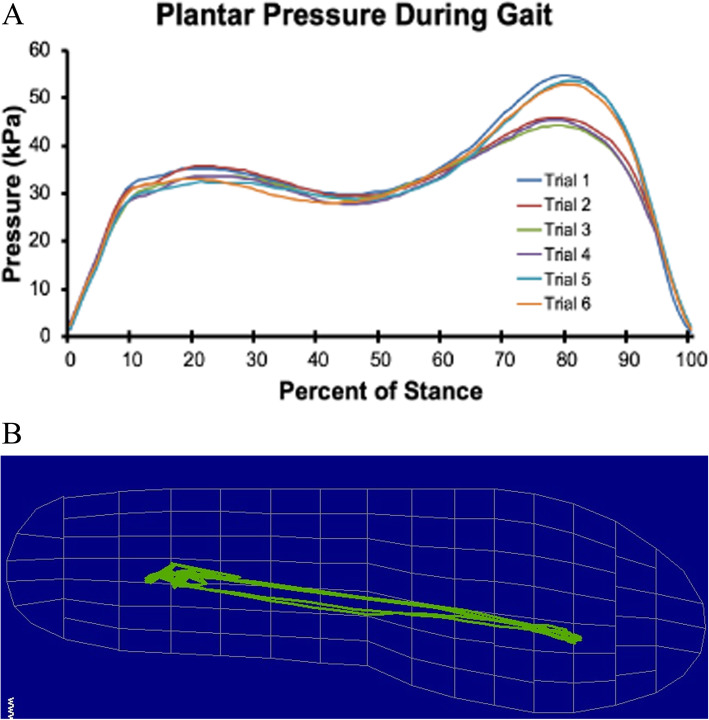
Plantar pressure measurements. **A** Total plantar pressure during the support phase of gait for 6 steps. **B** The path of the center of pressure during gait over 6 steps, superimposed on an outline of the foot

Pressure readings from each of the 99 discrete sensors will be used to calculate total plantar pressure (Fig. 13A) and the path of the center of pressure for each step (Fig. 13B). The peak plantar pressure from foot strike to midstance and the medial-lateral and anterior-posterior excursion of the center of pressure during support will be included in the aim 2 analysis.

##### Tissue stiffness

The average tissue stiffness for each of the four regions of the residual limb will be included in the aim 2 analysis.

##### Body kinematics

Trunk lean and hip flexion/extension during gait will be calculated from the conventional motion capture system (marker-based) for at least 6 steps per trial of treadmill walking. Body angles will be calculated using markers placed on the shoulders, C7, hips (ASIS and PSIS), greater trochanters, knee (or flexion axis of the prosthesis), ankle, and foot. We will determine the average of peak trunk lean and the average peak hip flexion and extension over all steps and all trials for each prosthesis. The average values will be included in the aim 2 analysis. Qualitative gait analysis will be performed by 3 investigators using the webcam videos to classify the participant’s gait asymmetry in terms of magnitude and timing.

##### Static radiographs

The distance from the most distal point of the residual femur to the inside surface of the socket under each weight-bearing condition will be measured using the freely available ImageJ software. Measurements will be performed by 3 researchers independently, with the middle value of the three measurements used for aim 2 analysis.

##### Hip strength

Peak hip strength and ROM will be recorded directly from the hand-held dynamometer and the goniometer. Strength and ROM will be measured three times, with the middle value of the three measurements used for aim 2 analysis.

#### Plans to promote participant retention and complete follow-up {18b}

Data collection protocols are designed to have a low burden of participation. For aims 1 and 2, active participation will entail one or two appointments at the research lab (after having been cast and fitted for a prosthetic socket). For aim 3, participation requires a small number of follow-up visits and is otherwise compatible with participants’ everyday life without restrictions. The reimbursement schedule follows the data collection/follow-up schedule to encourage the completion of the program.

#### Data management {19}

Data will be managed by the study statistician, who will work with the Biodynamics Lab staff to create the web-based application for data entry in collaboration with the study PIs. The statistician will also be responsible for monitoring data quality as the study is ongoing and merging any data not directly entered into a combined database for the study. Additional details related to the data management plan can be found in the Data Management section of this application.

#### Confidentiality {27}

The research lab is in an access-controlled building to which only research personnel have access. No sensitive or confidential information will be collected. All collected data will be de-identified and stored under a study ID, with the only document linking this ID to participant information being the consent form, which will be stored in a separate location from the data files. Electronic data storage complies with all applicable cybersecurity guidelines to prevent unauthorized access.

#### Plans for collection, laboratory evaluation, and storage of biological specimens for genetic or molecular analysis in this trial/future use {33}

No biological specimens will be obtained or stored for this study.

## Statistical methods

### Statistical methods for primary and secondary outcomes {20a}

#### Aim 1

The effects of in-socket mechanics, such as residual bone motion, peak skin strain, and pressure distribution on both physical function and patient-reported outcomes will be assessed using a generalized linear model (GLM). We will examine the effect(s) of each in-socket mechanical parameter on outcomes by utilizing the type III sum of squares tables. If our sample size drops below 30, we will develop separate models for each of the parameters of interest and outcome. Finally, we will create a unified model, containing the statistically significant parameter coefficients, which will allow us to predict physical function and patient response based on the changes to the included parameter estimates.

#### Aim 2

We will use partial correlation to examine the association between residual femur motion, skin strain, pressure, and readily available clinical measurements, while adjusting for potential confounders such as residual femur length (derived from CT data), overall residual limb length (measured from ischial tuber to distal end of the limb by caliper), contraction potential (tape measured as the difference of circumferential volumes during thigh muscle contraction and relaxation), and soft-tissue coverage (measured as a ratio between thigh and femur diameter). Partial correlation is a measure of the strength and direction of a linear relationship between two continuous variables whilst controlling for the effect of one or more other continuous variables (also known as “covariates” or “control” variables). Although partial correlation does not make the distinction between independent and dependent variables, the two variables are often considered in such a manner (i.e., you have one continuous dependent variable and one continuous independent variable, as well as one or more continuous control variables). If data is not approximately normal, we will use log transformations. Moderate-to-strong, significant correlations between research-grade and clinical data will be retained for empirical testing in aim 3.

#### Aim 3

Statistical analysis for aim 3 will involve two parts. First, the predictive model developed in aims 1 and 2 will be tested using data from 15 new participants (i.e., data not used to create the model). Second, we will compare the quantitative socket design to the current standard of care, in terms of patient outcomes and cost.

As part of the pilot clinical trial, we will use empirical data to test the socket modification model(s) created in aim 1 and clinical measure correlations obtained in aim 2. When empirical data are used to evaluate the model, there are several conventional statistical tests that can be applied to test the null hypothesis that the model output is wrong. These involve showing that some form of the predicted value does not equal the same form of the observed value (e.g., typical predicted, typical observed). The simplest empirical comparison is that of a regression of observed values on predicted values. We will obtain data as described for aims 1 and 2 on an additional 15 transfemoral prosthesis users using their current sockets and the sockets designed as part of the standard of care and through the new quantitative design process.

In order to compare the two methods of socket design (the quantitative design method compared to Standard of Care), we will compare patient-reported outcomes and cost between the two methods, utilizing the Wilcoxon-Mann-Whitney non-parametric test. Socket Comfort Scores (SCS, a simple patient-reported comfort score from 0 to 10) will be used to evaluate patient outcomes after wearing the two sockets. The SCS are reported to have a standard deviation of 2.3 points [50], meaning we will be powered to detect a difference of 1.1 points in the SCS given our sample size. With regard to costs, we will compare the two design approaches on the number of visits, cost of materials, and total cost of labor needed to develop the new socket. These costs will be recorded by the study prosthetist in his practice for the standard of care and in the Biodynamics Lab for the quantitative design method.

### Interim analyses {21b}

Due to sample size considerations, planned interim analyses will be descriptive in nature and will occur after our recruitment reaches 10 subjects. These statistics will include measures of central tendency (means, medians) and dispersion (standard deviations, interquartile range) for clinical measurements, socket mechanics, peak skin strain, and pressure distribution on both physical function and patient-reported outcomes. Data visualizations (line, bar graphs) will also be used to monitor the socket optimization process.

### Methods for additional analyses (e.g., subgroup analyses) {20b}

Not applicable, as no additional analyses are part of the protocol.

### Methods in analysis to handle protocol non-adherence and any statistical methods to handle missing data {20c}

Sensitivity analysis will be conducted with imputed values assuming missingness at random and nonignorable missingness.

### Plans to give access to the full protocol, participant-level data, and statistical code {31c}

The data sets analyzed during the current study and statistical code are available from the corresponding author on reasonable request, as is the full protocol.

### Oversight and monitoring

#### Composition of the coordinating center and trial steering committee {5d}

The study PI will monitor the study procedures designed to protect the privacy of participants and the confidentiality of the research data throughout the study. Participants will be identified by a numbering system known only to the study researchers. When data is recorded, only the participant ID number will be used for identification. Information that links the ID number given to each participant and that participant’s name or other personal information will be stored in a locked cabinet within the Biodynamics Lab and will be accessible only to the PI and lab manager. No individual participant information will be revealed after study participation or with the publication of results. The PI will conduct a regular review of accrued research data and other relevant information so as to ensure the validity and integrity of the data and to assure there is no change to the anticipated benefit-to-risk ratio of study participation. He will review radiographic data from the biplane X-ray system after each data collection trial to assure the quality of data collected is adequate to obtain the necessary measurements. Biplane radiography data will be monitored following collection and processing. This process is typically completed 1 to 2 weeks after the data collection session.

#### Composition of the data monitoring committee, its role, and reporting structure {21a}

Not applicable. A DMC is not required by the study sponsor.

#### Adverse event reporting and harms {22}

All participants will be told during the informed consent process that they should report any unpleasant side effects or adverse events to the study personnel, whether or not they feel the symptoms are related to the study care. The informed consent document will contain written instructions for reporting adverse events directly to the PI or lab manager, with all necessary contact information (email and phone). This will ensure that participants are not inhibited from informing the PIs or lab manager of any side effects or adverse events. Any adverse events that occur during patient testing will be immediately analyzed to determine if a change is necessary to the anticipated benefit-to-risk ratio of study participation and to determine whether the study should continue as designed, be changed, or be terminated. All investigators will monitor the current literature for related studies that may have an impact on the safety of study participants or the ethics of this research study. The PI will conduct an ongoing review of study procedures so as to ensure that the privacy of research participants and the confidentiality of their research data have not been violated. Upon receiving a report of unpleasant side effects or adverse events, the lab manager will first contact the participant directly to gather more information. The co-PIs will decide if the situation meets the University of Pittsburgh IRB definition of adverse event and whether it is related to the research protocol. Reporting of true adverse events in the context of the proposed program of research will occur according to the following University of Pittsburgh IRB definitions and timelines: Internal adverse events are adverse events that occur at a site that falls directly under the authority of the University IRB. Internal adverse events which are unexpected, fatal, or life-threatening and related or possibly related to the research must be reported to the IRB within 24 h of learning of the event. All other internal adverse events will be reported to the IRB within 10 working days of the investigator’s learning of the event.

#### Frequency and plans for auditing trial conduct {23}

Der is no predetermined frequency for trial auditing. The internal review committee and/or the sponsor may audit the trial at any point in time on short notice.

#### Plans for communicating important protocol amendments to relevant parties (e.g., trial participants, ethical committees) {25}

Any important protocol amendments will be submitted for approval to the institution’s IRB as well as the sponsor’s HRPO prior to implementing them. These entities will decide whether the changes need to be communicated to participants who have already completed the protocol priorly. Informed consent forms and processes will be updated accordingly, as will the study entry in the clinicalrials.gov database.

#### Dissemination plans {31a}

There are no restrictions on the publication of study results. They will be disseminated as conference abstracts and journal manuscripts, and de-identified data may be shared with other researchers upon individual request following the conclusion of the study.

## Discussion

Participants will receive radiation exposure beyond the amount they would have received had they not participated in this study. The additional radiation exposure includes testing within the biplane radiography system in the Biodynamics Labanda CT scan. Using commercial software (PCXMC; STUK, Helsinki, Finland), given the radiographic parameters required for this study, the effective radiation dose due to dynamic biplane radiography is estimated to be 2.0 mSv or less. For comparison, in the USA we receive about 3.0 mSv of exposure from natural background radiation every year, and the average effective dose associated with knee CT scans from our previous studies is 1.4 mSv. The Biodynamics Lab has had several knee-related research protocols approved by Radiation Safety and our Institutional Review Board (IRB). Those studies have been classified by the IRB as low-risk, even though they have had effective exposures similar to the proposed study. There is no minimum amount of radiation exposure that is recognized as being totally free of the risk of causing genetic mutations or cancer. However, the risk associated with the amount of radiation exposure that subjects will receive from taking part in this study is felt to be low and comparable to everyday risks. Subjects will be asked to perform maximal contractions during strength testing. This may cause temporary muscular discomfort.

Participants will be instructed on how to safely provide a urine sample. If the participant objects to performing the tasks associated with this study, the testing for that participant will be terminated immediately and the participant will no longer be a participant in the study. Participant safety will be continually monitored during data collection procedures. X-ray generators used in the Biodynamics Lab are FDA-approved for clinical use. All radiation safety devices built into the radiography system control console will remain fully functional at all times to ensure that the delivered radiation does not exceed the desired intensity or duration. Safety railings that can be used for support will be in place during all walking trials on the treadmill. The treadmill is also equipped with an emergency stop button that can be pushed to immediately stop the treadmill at any time. The research technician remains in the laboratory with the participant during all test procedures and can provide immediate assistance to the participant.

## Trial status

Protocol version 1.0

Recruitment start date: September 2021

Recruitment completion date (estimate): December 2023

## Abbreviations

3D: Three-dimensional
MRI: Magnetic resonance imaging
CT: Computerized tomography
ROM: Range of motion
VA: United States Department of Veterans Affairs
CAT-CAM: Contoured Adducted Trochanteric-Controlled Alignment Method
MAS: Marlo Anatomical Socket
NUFlex: Northwestern University Flexible [Socket]
MWK: Milwaukee [Socket]
HiFi: High Fidelity [Socket]
PBSS: Pohlig Bionic Socket System
